# Acute, isolated and unstable syndesmotic injuries are frequently associated with intra-articular pathologies

**DOI:** 10.1007/s00167-020-06141-y

**Published:** 2020-07-29

**Authors:** Kathrin Rellensmann, Cyrus Behzadi, John Usseglio, James Turner Vosseller, Wolfgang Böcker, Hans Polzer, Sebastian Felix Baumbach

**Affiliations:** 1 Department of General, Trauma and Reconstructive Surgery, University Hospital, LMU Munich, Nussbaumstraße 20, 80336 Munich, Germany; 2 Radiologie München, Dienerstraße 12, 80331 Munich, Germany; 3 Long Health Sciences Library, Columbia University Irving Medical Center, New York, USA; 4 Department of Orthopaedic Surgery, Columbia University Medical Center, New York, NY USA

**Keywords:** Syndesmotic injury, Syndesmosis, High ankle sprains, Arthroscopy, Chondral lesions, Surgery

## Abstract

**Purpose:**

Although simultaneous arthroscopy for the surgical treatment of acute isolated, unstable syndesmotic injuries has been recommended, little knowledge is present about the actual frequency of intra-articular pathologies for this injury. The aim of this study was to investigate the frequency and severity of intra-articular pathologies detected during arthroscopy and their subsequent treatment in acute isolated, unstable syndesmotic injuries.

**Methods:**

A retrospective chart review of patients treated by arthroscopic-assisted stabilization for acute isolated, syndesmotic instability was performed. The primary outcome parameter was the frequency of intra-articular pathologies. Secondary outcome parameters were the type of syndesmotic lesion (ligamentous/bony), severity of chondral lesions, MRI findings, treatment details, complications and the identification of factors associated with intra-articular pathologies.

**Results:**

Twenty-seven patients, 19% female, with a mean age of 37 ± 12 years met the inclusion criteria. 70% suffered isolated ligamentous injuries, the remaining suffered avulsion fractures of the syndesmosis. Chondral lesions occurred in 48% (ICRS grade II: 33%; ICRS grade IV 15%) and intra-articular loose bodies in 11% of patients. Overall, arthroscopy revealed intra-articular pathologies necessitating further treatment in 19% of patients. Neither the type of syndesmotic injury (bony vs. ligamentous; ns) nor the degree of ligamentous instability (West Point IIB vs. III; ns) had a significant influence on the occurrence of chondral lesions. One complication (SSI) occurred. Pre-operative MRI revealed a sensitivity/specificity of 100/79% for chondral lesions and 50/93% for loose bodies.

**Conclusion:**

Intra-articular pathologies in acute isolated, unstable syndesmotic injuries occur in up to 50% of patients, 19% necessitated additional treatment. Simultaneous arthroscopy, independent of the pre-operative MRI findings, appears reasonable in highly active patients.

**Level of evidence:**

Level III.

**Electronic supplementary material:**

The online version of this article (10.1007/s00167-020-06141-y) contains supplementary material, which is available to authorized users.

## Introduction

Isolated lesions of the syndesmosis, i.e. “high ankle sprains”, contribute only 1–17% of all ankle sprains in the general population [19, 21]. Yet, in high impact sports, they make up for up to 30% of all ankle sprains [25]. Multiple different classification systems for isolated syndesmotic injuries have been published [37], most of which differentiate acute (3–4 weeks), from subacute (to 12 weeks) and chronic injuries [13, 27], as well as stable and unstable injuries. The stability of syndesmotic injuries most often is assessed by the West Point Ankle Grading System [19, 37] in its adaptation from Calder et al. [6]. Most authors agree that grade IIB and III lesions necessitate surgical stabilization [6]. Today, surgical treatment consists of closed reduction of the distal tibiofibular joint and indirect fixation, either static or dynamic. Although static fixation by means of one or two syndesmotic screws is considered to be the most common procedure, emerging evidence points at a superior patient reported outcome for the dynamic fixation by a suture button system [31].

It is worth noting that accompanying intra-articular pathologies, such as loose bodies and chondral lesions, have been reported in up to 30% of patients suffering chronic lateral ankle instability [7, 15, 22] and in up to 80% of patients with ankle fractures [4, 20]. To identify and treat these pathologies, additional arthroscopy is recommended during the surgical treatment of both, chronic ankle instability [23, 39] and complex ankle fractures [34, 35]. Magnetic resonance imaging (MRI) is often considered a non-invasive alternative to pre-operatively identify patients with intra-articular pathologies. Still, MRI has been reported to have a limited sensitivity in detecting chondral lesion and loose bodies [2, 18, 26, 30]. Arthroscopy on the other hand does not only allow to reliably identify and treat intra-articular pathologies but also seems to improve the patient reported outcomes [23, 34, 35, 39]. Considering that syndesmotic injuries result from a comparable injury mechanism, i.e. ankle sprains, it can be suspected that intra-articular pathologies also occur frequently in patients suffering an unstable syndesmotic injury. Particularly because syndesmotic injuries occur predominantly in a physically highly active population, it seems reasonable that these patients would benefit from arthroscopic-assisted surgery.

In the course of this study, we conducted a thorough literature review to identify studies reporting on intra-articular pathologies in patients treated for an acute isolated, unstable syndesmotic injury. Although various authors have recommended arthroscopy [11, 14, 38], only one study mentioned chondral lesions detected by arthroscopy. Unfortunately, these were only reported as a side note, and neither their severity nor their treatment was documented [8]. Consequently, no study has yet in detail assessed the frequency of intra-articular pathologies in patients suffering an acute isolated, unstable syndesmotic injury.

The authors hypothesized that intra-articular lesions are common in patients suffering an acute isolated, unstable syndesmotic injury. Up to date, the frequency of accompanying intra-articular lesions in acute isolated, unstable syndesmotic injury is unknown. Therefore, the purpose of this study was to investigate the frequency and severity of intra-articular pathologies detected during arthroscopy and their subsequent treatment in acute isolated, unstable syndesmotic injuries.

## Materials and methods

After receiving approval from the ethics committee of the University Hospital, LMU Munich (IRB ID: 19-868), a retrospective chart review was performed to identify patients treated by arthroscopic-assisted stabilization of acute syndesmotic instability.

### Patient cohort

A retrospective chart review was conducted based on the ICD-10: S93.2 as well as OPS-2019: 5–869.2 and 1–697.8 between 01/2014 and 09/2019. Inclusion criteria were patient age ≥ 18 years and arthroscopic-assisted surgical treatment of acute isolated, unstable syndesmotic injuries. An injury was considered acute if it was treated within the first 12 weeks after trauma [28, 32, 36]. Isolated syndesmotic injuries were defined as ligamentous injuries and/or syndesmotic avulsion fractures, i.e. fracture to the posterior malleolus, Tubercule de Tillaux Chaput or Wagstaff fragment [3]. Unstable was defined as a two-ligament lesion with instability proven during arthroscopy or functional radiographic examination. Syndesmotic instability, at our department, is assessed using radiograph, MRI, CT and, in case of uncertainty, an “external rotation test” under fluoroscopy [17] is performed. Instability was defined as either dislocated bony avulsion of the syndesmotic complex (posterior malleolus, Tubercule de Tillaux Chaput, or Wagstaff fragment) or positive external rotation test under fluoroscopy in pure ligamentous injuries.

### Treatment

Our Foot and Ankle Division is part of the Department of General, Trauma and Reconstructive Surgery. Depending on the paths of admission, patients are either seen by the trauma surgeon on call or referred to our outpatient clinic. Starting in mid-2014, patients suffering an acute isolated, unstable syndesmotic injury presenting to the Division of Foot and Ankle Division were routinely treated by additional arthroscopy. Arthroscopy was first performed utilizing the standard anteromedial and -lateral portals. No traction device was applied. Impinging soft tissue and loose bodies were removed. Then the joint was explored, and cartilage lesions graded according to the recommendations of the International Cartilage Repair Society (ICRS; [5]). In case of ICRS grade II and III lesions, chondroplasty was performed per the choice of the surgeon. ICRS grade IV lesions were all treated by micro-/nanofracturing. Non-dislocated PM fragments of sufficient size were fixed percutaneously by AP screws and dislocated ventral bony syndesmotic avulsion (Tubercule de Tillaux Chaput, or Wagstaff fragment) by open reduction and internal fixation. The distal tibiofibular joint was stabilized by suture button(s) (TightRope, Fa. Arthrex, Naples, FL, USA) ± a syndesmotic screw or internal brace, per the surgeon’s preference. Patients admitted to our Trauma Division but not to the Division of Foot and Ankle Surgery, were all treated by a fellowship trained trauma surgeon. They utilized the same protocol to treat bony and ligamentous syndesmotic injuries. However, no additional arthroscopy was performed and, therefore, these patients were excluded.

### Primary and secondary outcome parameters

The primary outcome parameter was the incidence of intra-articular pathologies, i.e. chondral lesions, loose bodies or osteochondral lesions, detected during arthroscopy. Secondary outcome parameters were the severity of the cartilage lesions according to the ICRS grading system [5], its localization per the anatomical grid scheme [12], treatment details including treatment of the cartilage lesion, fixation of avulsion fractures, type of syndesmotic stabilization and complications related to the additional arthroscopy. In addition, all available preoperative MRI scans were anonymized and reviewed by a blinded senior radiologist with special interest in musculoskeletal and cartilage imaging. In concordance with the arthroscopic grading, the severity and localization of the cartilage lesions were rated per the ICRS grading system [5] and the anatomical grid scheme [12], respectively. Any intra-articular loose body was documented. For image analysis, a standard post-processing workstation was used in all data sets. The blinded radiological findings were then compared to the intra-operative findings.

### Statistical analysis

Standard descriptive statistics, independent t test, and Chi square tests were performed using SPSS (Version 25, IBM, Armonk, United States). If not stated differently, values are given a mean ± SD. The level of significance was set to *p* < 0.05.

## Results

**Fig. 1:**
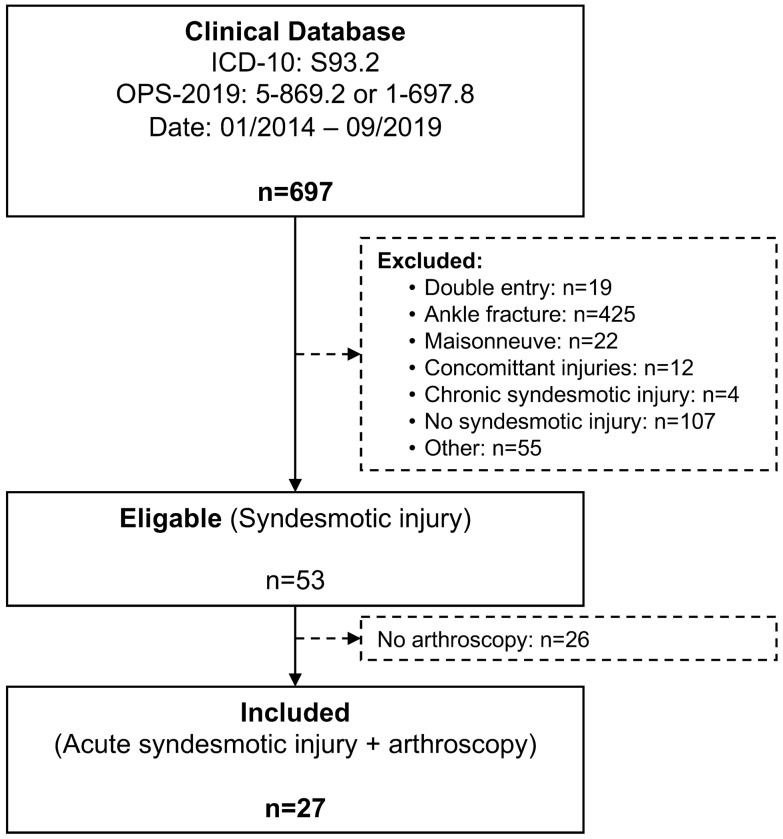
Flow-chart illustrating the patient selection process. ICD-10 S93.2: Traumatic rupture of ligaments of the ankle or foot; OPS 5-869.2: Stabilization of syndesmotic lesions with fixation methods; OPS 1-697.8: Diagnostic arthroscopy of the upper ankle joint; Concomitant injuries: Fractures of the tibial shaft or metatarsal fractures; Other: Ligamentous injury of the elbow or knee

The database search yielded 697 results. Out of these, 53 patients suffered an acute, isolated, unstable syndesmotic injury. Twenty-seven of which were treated with additional arthroscopy (Fig. 1). The demographic details including the ASA score, trauma mechanism and injury type are outlined in Table 1, as well as a comparison to the excluded non-arthroscopic cohort. Both cohorts showed no significant differences, but significantly more bony avulsion syndesmotic lesion were found in the arthroscopy cohort (*p* = 0.024).

**Table 1:** Comparing the included arthroscopy cohort to the excluded non-arthroscopy cohort

	Arthroscopy cohort (*n* = 27)	Non-arthroscopy cohort—excluded (*n* = 26)	*p* value
Age (years)	37 ± 12	34 ± 16	ns
% Female	19%	41%	ns
ASA score	1.9 ± 0.6	1.6 ± 0.7	ns
Trauma mechanism			
Ankle sprain	82%	90%	ns
Fall from height < 1 m	14%	5%	
High energy trauma	4%	5%	
Isolated ligamentous syndesmotic injury	67% (Westpoint IIB: 48%; Westpoint III: 19%)	95% (Westpoint IIB: 81%; Westpoint III: 38%)	0.024
Bony avulsion syndesmotic lesions	33%	5%	

ASA, American Society of Anesthesiologists Classification; m, meter; n, number

**Fig. 2:**
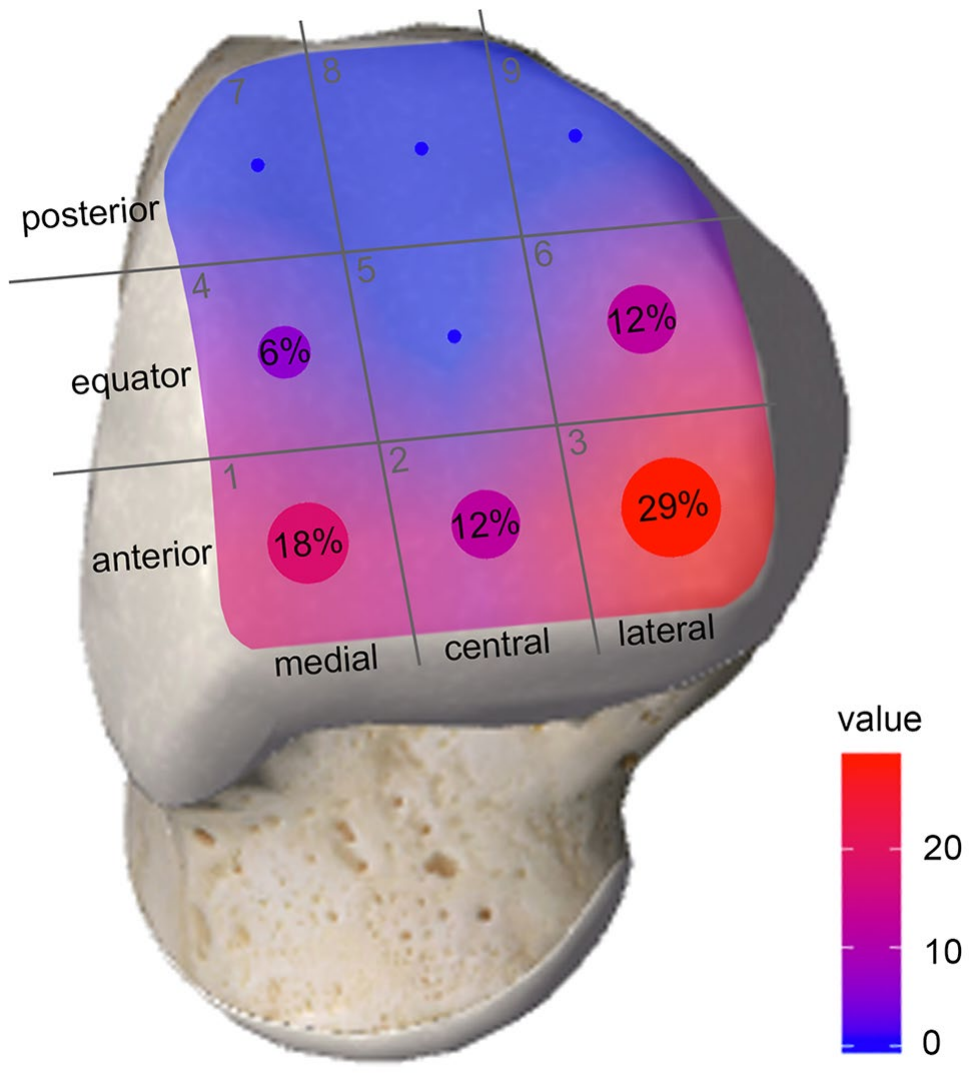
Illustration of the localization of the talar chondral lesions per the anatomical grid scheme

The detailed injury patterns and treatment details per patient are shown in supplement 1. Per the primary outcome parameter, 15 chondral lesions occurred in 13 (48%) patients. In four patients, there occurred five (15%) ICRS grade IV lesions, which necessitated treatment by micro-/nanofracturing. No osteochondral lesions were observed. 76% of cartilage lesion were talar (Fig. 2), 24% tibial.

Loose bodies were detected and removed in 3 (11%) patients, two of which also had an ICRS grade IV chondral lesion. Neither the type of syndesmotic injury (bony vs. ligamentous; ns) nor the degree of ligamentous instability (West Point IIB vs. III; ns) had a significant influence on the occurrence of chondral lesions. ICRS grade IV lesions occurred in 2 out of 18 (11%) patients with an isolated ligamentous syndesmotic injury and in 2 out of 9 (22%) patients with a bony avulsion (ns). Overall, five (19%) patients presented with an intra-articular lesion (four patients with chondral injuries grade IV, one patient with loose bodies) necessitating additional treatment. One complication (4%), i.e. surgical site infection (SSI) with revision surgery of the anterior-lateral portal, was observed in the herein presented cohort.

Pre-operative MRIs were available for review in 17 patients (74%). As most of the MRI images had not been conducted at our hospital, their quality varied considerable. Overall, MRIs were generated on 9 different scanners, 71% of which were 1.5 Tesla, the remaining 3 Tesla devices. Based on a four-item Likert scale (1 very good, 4 poor), the MRI image quality was overall rated as good (2.0 ± 0.9). The MRI review resulted in six chondral lesions ICRS grade IV (35% of patients), two lesions ICRS grade I to III (12% of patients), and two loose bodies (12% patients). Overall, in 35% of patients, arthroscopy would have been indicated based on the MRI findings (chondral lesion ICRS grade IV ± loose bodies). Based on the four-fold table in Table 2, MRI revealed a sensitivity/specificity of 100/79% for chondral lesions and 50/93% for loose bodies. The positive predictive/negative predictive value for chondral lesions were 0.5/1 and 0.5/0.93 for loose bodies.

**Table 2:** Four-fold table of MRI findings on chondral lesions and loose bodies compared to the arthroscopic findings

	ICRS IV	ICRS ≤ III	Loose bodies	No loose bodies
Positive	3	3	1	1
Negative	0	11	1	14

## Discussion

The aim of this study was to investigate the frequency and severity of intra-articular pathologies detected during arthroscopy and their subsequent treatment in acute isolated, unstable syndesmotic injuries. Twenty-seven patients were retrospectively reviewed and arthroscopy revealed chondral lesions in 48% and loose bodies in 11%. Overall, 19% of patients presented with intra-articular pathologies necessitated additional treatment, i.e. micro-/nanofracturing and/or removal of loose bodies. On the contrary, pre-operative MRI revealed chondral lesions in 35% and loose bodies in 12% of the patients. Based on the pre-operative MRI findings, additional arthroscopy would have been indicated in 35% of the patients.

As stated in the introduction, we initially conducted a thorough literature review. Only one study on arthroscopically detected intra-articular pathologies during surgical treatment of acute isolated, unstable syndesmotic injuries was identified. D’Hooghe et al. [8] presented a retrospective study on 110 male professional football players who underwent arthroscopic assisted treatment for acute (< 6 weeks), isolated unstable (West Point grade ≥ IIB) syndesmosis injuries. Their primary outcome was sport-specific rehabilitation, but cartilage injuries were also noted, which occurred in 21% of the injured ankles. They occurred significantly more often in West Point grade III compared to IIB injuries (40% vs. 12% *p* = 0.0019). A multivariable analysis revealed that injury severity and the presence of a talar cartilage injury were significant negative predictors for return to sport-specific rehabilitation (*p* < 0.001/*p* = 0.023), return to team training (*p* < 0.001/*p* = 0.01) and return to match play (*p* < 0.001/*p* = 0.031). Unfortunately, concomitant intra-articular pathologies were only a secondary outcome parameter, and therefore, neither the severity of the chondral lesions, nor the subsequent treatment was reported. This lack of literature highlights the necessity for more detailed studies on intra-articular pathologies in acute isolated, unstable syndesmotic injury.

In the herein presented patient cohort, almost 50% of patients suffered intra-articular lesions. More importantly, 19% of patients required additional treatment because of intra-articular lesions. In contrast to the study by D’Hooghe et al. [8], neither the type of syndesmotic injury (bony vs. ligamentous; ns) nor the degree of ligamentous instability (West Point IIB vs. III; ns) had a significant influence on the occurrence of chondral lesions. This could be explained by the limited sample size. Still, the herein presented study highlights for the first time, the high frequency of intra-articular pathologies in detail for acute isolated, unstable syndesmotic injuries, reports on the subsequent treatments and compares their frequency to pre-operative MRI findings.

MRI is the current standard diagnostic tool to identify intra-articular lesions pre-operatively. In the current study, pre-operative MRI had a sensitivity/specificity of 100/79% for chondral lesions and 50/93% for loose bodies. The herein observed sensitivity compares favorably to previous studies with reported sensitivity rates ranging from 19 to 71% [2, 18, 26, 30]. This high rate could be explained either by the rather small cohort or the experience of the senior radiologist reviewing the images especially for intra-articular pathologies. The sensitivity of 50% for loose bodies compared well to a previous study by O’Neill et al. [26]. The positive- (0.5/0.5) and negative predictive values (1/0.93) for chondral lesions/loose bodies again compared well to the literature [18]. In our particular case, 6 of 17 patients (35%) would have classified for additional arthroscopy based on the pre-operative MRIs (ICRS grad IV ± loose bodies). Although MRI did detect all ICRS grade IV chondral lesions (100%), one patient with an intraarticular body was missed (50%). In three patients the MRI was false positive.

In accordance with the present literature, these findings underline the higher diagnostic accuracy for arthroscopy compared to MRI in detecting intra-articular lesions [24, 33]. Still, additional arthroscopy bears the risk for additional complications. In our cohort there was one instance of surgical site infection (4%). A recent comprehensive literature review analyzed the complications following anterior and posterior ankle arthroscopy based on 55 studies [41], six of which included more than 100 patients [1, 9, 16, 29, 40, 42]. These six studies reported a cumulative complication rate of 4.9% in 2676 patients. Lesions to the Superficial Peroneal Nerve occurred in 1.7%, surgical site infections including sinus tract formation in 1.5%, other nerve injuries in 1.3%, deep vein thrombosis in 0.1%, vascular injuries in 0.04%, and other complications in 0.3% of patients. In summary, ankle arthroscopy appears to have a limited complication rate of about 5%.

The key question for our clinical practice, therefore, is whether to base the indication for additional arthroscopy on pre-operative MRI, or to perform additional arthroscopy in all patients suffering an acute isolated, unstable syndesmotic? If the pre-operative MRI is chosen, there is a risk to miss a percentage of patients with intra-articular pathologies that would have necessitated treatment. On the other hand, treating all patients with additional arthroscopy might increase the total number of complications. We believe, that for now, the data available is not sufficient to answer this complex question comprehensively. This is predominantly because the influence of additional arthroscopy on the subjective, patient reported outcome is unknown for acute isolated, unstable syndesmotic injuries.

Following this study, our treatment strategy was adapted. For patients with high physical demands, additional arthroscopy is recommended, independent of the pre-operative MRI. In older patients with moderate level of activity, treatment recommendation are based on the preoperative MRI. This is done for several reason: first, micro-/nanofracturing has been shown to result in a superior outcome compared to debridement only for full-thickness cartilage lesions [10]; second, various studies on other pathologies, such as chronic ankle instability [23, 39] or complex ankle fractures [34, 35], were able to demonstrate that arthroscopic assisted surgery with immediate treatment of intra-articular pathologies seems superior to conventional (non-arthroscopic) treatment; third, D’Hooghe et al. [8] were able to show, that talar cartilage lesions were a significant negative predictor for return to sports in this cohort. Therefore, one should not risk to miss any intra-articular pathology in highly active patients and rather accept the risk for a complication.

The study has several limitations, which should be discussed. First of all, the retrospective design. Although MRIs were only available in 74% of patients, the primary outcome parameter, i.e. intra-articular pathologies detected by arthroscopy, were generated from the surgical reports, which were available for all patients. Therefore, the risk of bias due to the retrospective data assessment could be considered as low. A further limitation might have been a selection bias. Only patients seen through by the Foot and Ankle Division were treated with additional arthroscopy. Although this can be rated as a pseudo-randomization, one could assume, that more active patients were referred to our outpatient clinic. Still, although the type of injury (ligamentous vs. bony) varied significantly between the two cohorts, none of the further assessed parameters did. Finally, the overall sample size of 27 is rather small, but the largest published so far.

## Conclusion

Intra-articular pathologies occur in up to 50% of patients suffering an acute isolated, unstable syndesmotic injury. Nineteen percent of patients suffered ICRS grade IV lesions or had loose bodies that necessitated additional treatment. Whether the indication for additional arthroscopy should be based solely on pre-operative MRI remains unclear. For aged patients with a moderate level of activity, we base the indication for additional arthroscopy on the pre-operative MRI, due to its considerably high negative predictive value. In highly active patients, we routinely perform additional arthroscopy as we do not want to risk missing any intra-articular pathology and rather take the risk for a complication.

## Electronic supplementary material

Below is the link to the electronic supplementary material.

Supplementary material 1 (DOCX 21 kb)

## Abbreviations

CT: Computed tomography
ICRS: International Cartilage Repair Society
MRI: Magnetic resonance imaging
SSI: Surgical side infection
ns: Not significant
