# Supplementary material for: Acute, isolated and unstable syndesmotic injuries are frequently associated with intra-articular pathologies
Source: Knee Surg Sports Traumatol Arthrosc. 2020 Jul 29;29(5):1516–22. doi: 10.1007/s00167-020-06141-y (PMC8038950; doi:10.1007/s00167-020-06141-y)
Supplement: Supplementary file 1 — Supplementary material 1 (DOCX 21 kb) [file 167_2020_6141_MOESM1_ESM.docx]

| **Table 1.:** Patient details including intra-articular pathologies and treatment. | | | | | | | | | | | | |
| --- | --- | --- | --- | --- | --- | --- | --- | --- | --- | --- | --- | --- |
| **ID** | **Age**  **[a]** | **Sex** | **Injury pattern** | **Treatment syndesmosis** | **MRI**  n=17 (63%) | | | **Arthroscopy**  n=27 (100%) | | | |  |
|  |  |  |  |  | **Cartilage damage [ICRS]** | **Cartilage damage location** | **Loose bodies** | **Cartilage damage [ICRS]** | **Cartilage damage location** | **Cartilage damage treatment** | **Loose bodies** | **Comp** |
| 1 | 27 | F | Lig. synd.  Grade IIB | Suture button (1x) | - | - | - | II | 3 | Chondroplasty | No | None |
| 2 | 31 | F | Synd. + PM | Suture button (1x) | - | - | - | 0 | / | / | No | None |
| 3 | 29 | M | Lig. Synd  Grade IIB | Suture button (2x) | IV | 6 | No | 0 | / | / | No | None |
| 4 | 38 | M | Synd. + Tub. T.C. | Suture button (1x) ORIF screw Tub. T.C. | - | - | - | II | 11 | Chondroplasty | No | None |
| 5 | 49 | M | Lig. synd.  Grade IIB | Suture button (2x) | - | - | - | IV / IV* | 4 / 12 | Micro-/Nano fracturing | Yes | None |
| 6 | 35 | M | Lig. synd.  Grade III | Suture button + Synd. screw | - | - | - | 0 | / | / | No | None |
| 7 | 38 | M | Synd. + Tub. T.C. + PM | Suture button (2x) | III | 6 | No | 0 | / | / | Yes | None |
| 8 | 22 | M | Lig. synd.  Grade IIB | Suture button (2x) | - | - | - | 0 | / | / | No | None |
| 9 | 44 | M | Synd. + PM | Suture button (2x) | 0 | / | No | 0 | / | / | No | None |
| 10 | 24 | F | Lig. synd.  Grade IIB | Suture button (2x) | 0 | / | No | II | 3 | Chondroplasty | No | None |
| 11 | 42 | M | Lig. Synd. + lateral lig.  Grade IIB | Suture button (2x) Open Broström | III / IV | 6 / tibial | No | 0 | / | / | No | None |
| 12 | 29 | M | Synd. + PM | Suture button (1x) CRIF AP Screws PM | 0 | / | No | 0 | / | / | No | None |
| 13 | 52 | F | Lig. synd.  Grade III | Suture button (1x) | IV | 6 | No | II | 2 | Chondroplasty | No | None |
| 14 | 26 | F | Lig. synd.  Grade IIB | Suture button (2x) | 0 | / | No | II | 1 | None | No | None |
| 15 | 37 | M | Synd. + PM | Suture button (2x) CRIF AP Screws PM | IV | tibial | No | IV / II* | 3 / 1 | Micro-/Nano fracturing | No | None |
| 16 | 44 | M | Synd. + PM + Deltoid | Suture button (1x) Suture Deltoid | IV | 6 / tibial | Yes | IV | 3 / 6 | Micro-/Nano fracturing | No | None |
| 17 | 20 | M | Lig. synd.  Grade IIB | Suture button (1x) | 0 | / | No | 0 | / | / | No | None |
| 18 | 29 | M | Lig. synd.  Grade IIB | Suture button (2x) | 0 | / | No | 0 | / | / | No | None |
| 19 | 30 | M | Lig. synd.  Grade III | Suture button (2x) | 0 | / | No | II | 11 | None | No | None |
| 20 | 26 | M | Lig. synd.  Grade III | Suture button (1x) | 0 | / | No | II | 3 | Chondroplasty | No | None |
| 21 | 39 | M | Lig. synd.  Grade IIB | Suture button (1x) | - | - | - | 0 | / | / | No | None |
| 22 | 22 | M | Lig. synd.  Grade IIB | Suture button (2x) | 0 | / | No | II | 10 | Chondroplasty | No | None |
| 23 | 52 | M | Synd. + Tub. T.C. + PM | Suture button + Int. Brace | - | - | - | 0 | / | / | No | None |
| 24 | 30 | M | Lig. synd.  Grade IIB | Suture button + Int. Brace | - | - | - | 0 | / | / | No | None |
| 25 | 69 | M | Synd. + Tub. T.C. + PM | Suture button (1x) | - | - | - | 0 | / | / | No | None |
| 26 | 44 | M | Lig. synd.  Grade IIB | Suture button (2x) | 0 | / | No | II | 2 | Chondroplasty | No | None |
| 27 | 61 | M | Lig. synd.  Grade III | Suture button (1x) | IV | 6 | Yes | IV | 6 | Micro-/Nano fracturing | Yes | SSI |
| F: Female, M: Male; Lig. Synd.: Ligamentous syndesmotic rupture; Synd.: Syndesmosis; PM: Posterior malleolus; Tub. T.C.: Tubercule de Tillaux Chaput; ORIF: Open reduction and internal fixation; CRIF: Close reduction internal fixation; AP Screws: Anterior to posterior screws; Int. Brac: Internal brace; *: In the order as stated for cartilage damage location; SSI: Surgical side infection; /: not applicable  Cartilage damage location - 1: ant. med.; 2: ant. central; 3: ant. lat.; 4: mid. med.; 5: mid. central; 6: mid. lat.; 7: post. med.; 8: post. central; 9: post. lat.; 10: tibial med.; 11: tibial lat.; 12: tibial ventral | | | | | | | | | | | | |
